# Which Way to the Summit?

**DOI:** 10.19102/icrm.2020.111201

**Published:** 2020-12-15

**Authors:** Aniruddha Vyas, Yash Lokhandwala, Ankit Mahajan

**Affiliations:** ^1^Medanta Hospital, Indore, India; ^2^Holy Family Hospital and Research Center, Mumbai, India

**Keywords:** Ablation, great cardiac vein, idiopathic ventricular tachycardia, left ventricular summit

## Abstract

A 57-year-old man presented with palpitations and dizziness for one day. He reported a history of similar short-lasting, self-limiting episodes in the past. Evaluation showed a hemodynamically stable, ongoing monomorphic ventricular tachycardia (VT) with positive concordance in the precordial leads and inferior axis. A structurally normal heart was seen on echocardiography. The VT was cardioverted to normal sinus rhythm with a biphasic 100-J direct-conversion shock under mild sedation, only to spontaneously start over again. In view of the patient’s structurally normal heart, a previous history of similar complaints in the past, and no obvious trigger including ischemia for VT, he subsequently underwent an electrophysiology study (EPS).

## Case presentation

A 57-year-male presented with palpitations and lightheadedess for one day. An electrocardiogram (ECG) showed a wide complex tachycardia, while echocardiography revealed a structurally normal heart. He was taken for electrophysiology study (EPS). No antiarrhythmic drugs were given prior to the EPS. He was in ongoing hemodynamic stable ventricular tachycardia (VT) at the time of the EPS. A screening coronary angiogram showed normal epicardial coronary arteries.

**Figure 1:**
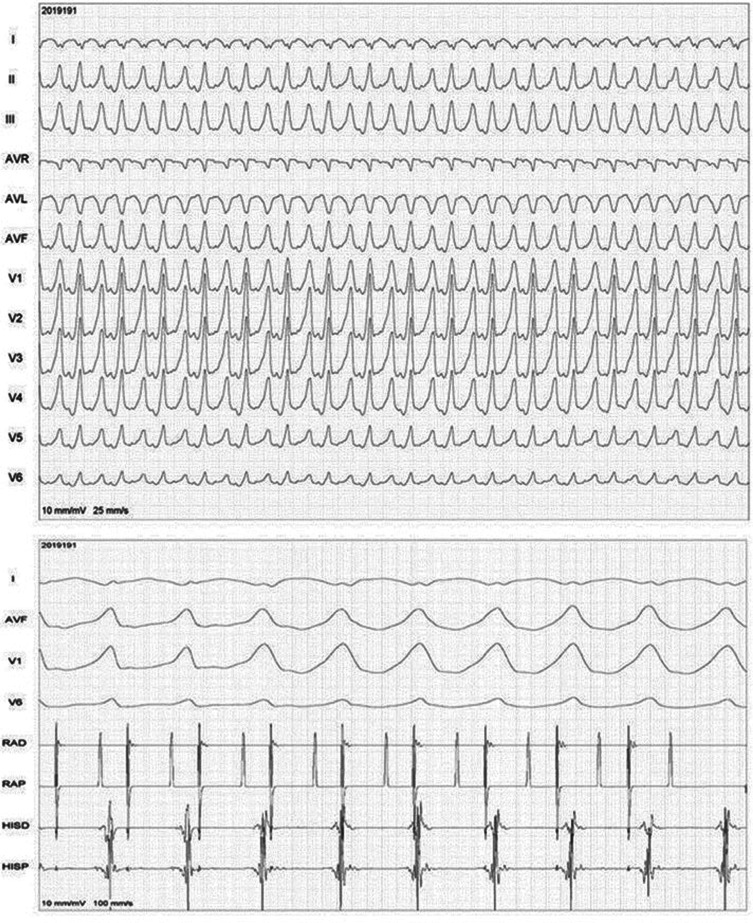

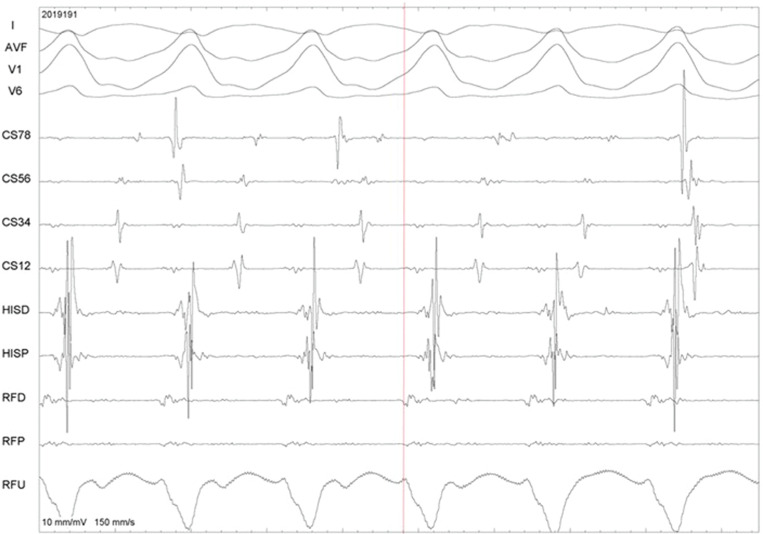
Upper panel: 12-lead ECG of tachycardia. Lower panel: rapid RA pacing during tachycardia. Presented are the surface ECG leads I, aVF, and V1 and intracardiac recordings. RAD: right atrial distal; RAP: right atrial proximal; HISD: His distal; HISP: His proximal.

**Figure 1** shows the 12-lead ECG during EP and the intracardiac recordings of rapid right atrial (RA) pacing during tachycardia.

**Figure 2:**
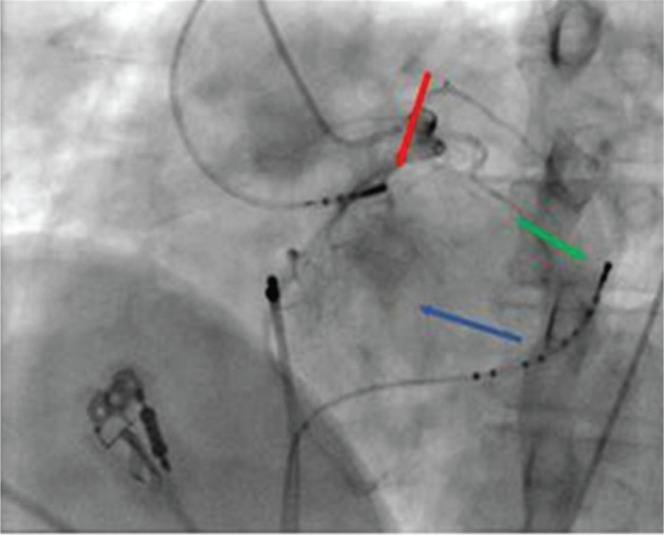
Upper panel: AV dissociation is apparent during VT. Signals at the left sinus of Valsalva and anterolateral mitral annulus (CS1,2) were noted relative to the surface QRS. Shown here are the surface ECG leads I, aVF, V1, and V6 and intracardiac recordings. CS: coronary sinus (higher numbers mean proximal electrodes and vice versa); RFD: ablation catheter distal; RFP: ablation catheter proximal; RFU: unipolar from the distal electrode of the ablation catheter. Lower panel: Corresponding sites on fluoroscopic images for left sinus of Valsalva (red arrow) and anterolateral mitral annulus (green arrow). Blue arrow points to the left ventricular outflow tract.

**Figure 2** shows the intracardiac recordings of signals at the left sinus of Valsalva and the anterolateral mitral annulus relative to the onset of surface QRS. Further, a fluoroscopic image of the left anterior oblique view reveals the relative positioning of catheters in the traces above.

**Figure 3:**
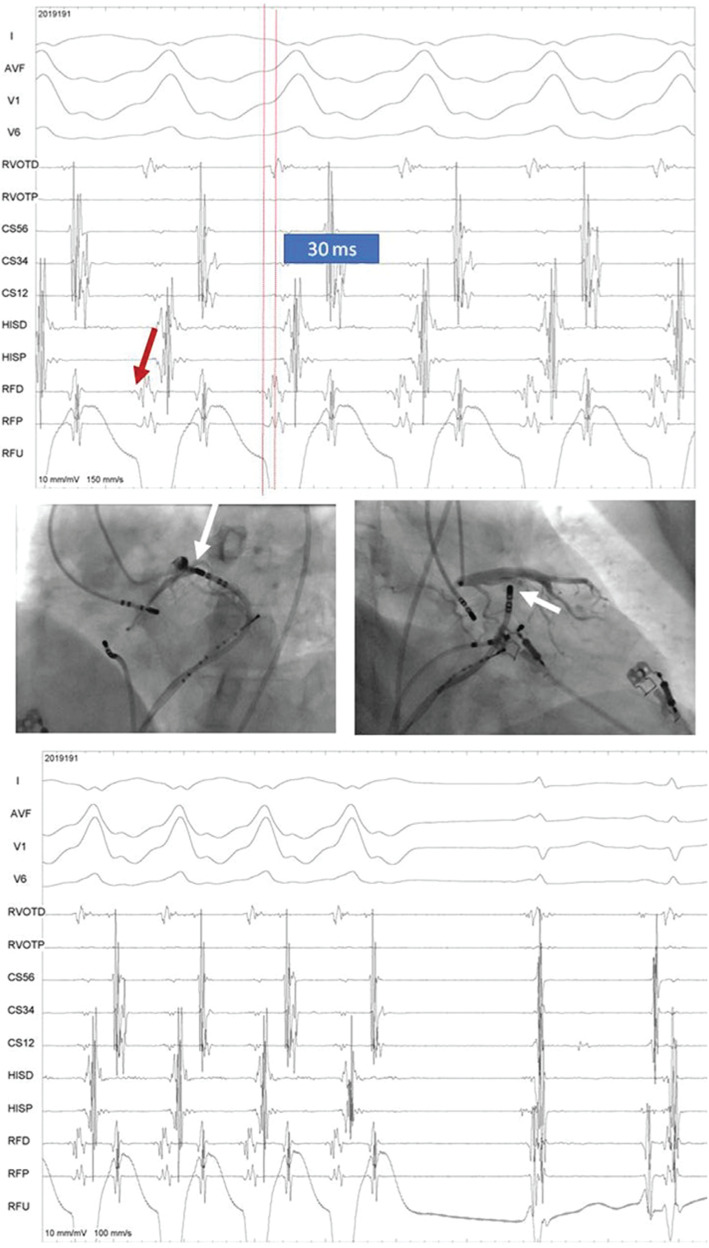
Upper panel: site of earliest local signals, 30 ms earlier than the surface QRS with distinct presystolic potential (red arrow) recorded at this site. Shown here are the surface ECG leads I, aVF, V1, and V6 and intracardiac recordings. Middle panel: fluoroscopic views in LAO 40° and RAO 30° angles with the ablation catheter (white arrow). Lower panel: during RF energy delivery. Shown are the surface ECG leads I, aVF, V1, and V6 and intracardiac recordings.

**Figure 3** shows the intracardiac recordings of signals at the site of successful ablation, fluoroscopic images in the left anterior oblique and right anterior oblique views, and the clean termination of tachycardia soon after the onset of radiofrequency (RF) energy with an irrigated-tip catheter.

## Discussion

A 12-lead ECG of the clinical tachycardia revealed a wide complex tachycardia at a rate of around 180 bpm with positive concordance in precordial leads, an inferior axis, and a 1:1 ventriculoatrial relationship. Overdrive RA pacing during tachycardia brought out the atrioventricular (AV) dissociation during tachycardia. Interestingly, the first two complexes in the lower panel of **Figure 1** during rapid RA pacing demonstrate fused complexes with a small His bundle signal evident in the His proximal channel. In view of R in V1 to V6 and the inferior axis on the 12-lead ECG, activation mapping [RF distal (RFD) channel in **Figure 2**, upper panel] was performed initially in the left sinus of Valsalva, which did not yield a good early signal (**Figure 2**, red bar in the upper panel and red arrow in the lower panel). Also, the corresponding unipolar [third dimension in conventional two-dimensional (2D) mapping] signal showed an initial positive wave. Signals at the anterolateral mitral annulus as seen in recordings in the distal coronary sinus channel (CS1, CS2) also suggested late activation (green arrow in the lower panel). However, as no other early site was observed in the left ventricular outflow tract, with difficulty, a 6-French ablation catheter was advanced deeper into the coronary sinus where early activation was seen. This was then exchanged for an irrigated-tip 7-French ablation catheter. As seen in the upper panel of **Figure 3**, the earliest local signals occurring about 30 ms earlier than surface QRS were apparent at the great cardiac vein at the left ventricular summit. The steerable irrigation-tip ablation catheter could be advanced with some maneuvering to the ablation site; a steerable long sheath may at times be required for this negotiation. Distinct small presystolic potentials were also seen at this site (red arrows in **Figure 3**, upper panel). Corresponding fluoroscopic images in left anterior oblique and right anterior oblique angulation with proximity to the coronary arteries is seen in the middle panel of **Figure 3**. Successful ablation by irrigation-tip catheter led to clean termination of the tachycardia at this site as seen in the lower panel of **Figure 3**. Settings of 20 W for power, 45°C for temperature, and 45 seconds for duration of RF energy application were used. No VT was inducible after this and the patient remained asymptomatic three months later. The mapping procedure was performed on a conventional 2D mapping system (EP Tracer; Schwarzer Cardiotek GmbH, Heilbronn, Germany) with mild sedation. Isoproterenol was not used during EPS.

